# Hematological and biochemical changes due to short-term oral administration of imidacloprid

**DOI:** 10.4103/0971-6580.75843

**Published:** 2011

**Authors:** Tarun Balani, Seema Agrawal, A. M. Thaker

**Affiliations:** Preclinical Department, Cadila Pharmaceuticals Ltd., Ahmedabad, India; 1 Department of Pharmacology and Toxicology, College of Veterinary Science and Animal Husbandry, Anand Agricultural University, Anand - 388 001, Gujarat, India

**Keywords:** Hypoglycemia, imidacloprid, leukopenia, serum glutamate oxaloacetate transaminase

## Abstract

Subacute toxicity of repeated (28 day) oral administration of imidacloprid in male White Leghorn (WLH) chicks was assessed. One hundred and twenty-five birds were divided into five groups, with each group containing 25 birds. The birds of group C1 were given no treatment and served as control. Group C2 was administered groundnut oil (1 ml/kg) and served as control (vehicle). Group I1 was given 1/40^th^ of apparent LD_50_ (ALD_50_) (1.25 mg/kg), and group I2 was put on 1/30^th^ of ALD_50_ (1.67 mg/kg), while group I3 received 1/20^th^ of ALD_50_ (2.5 mg/kg) of imidacloprid suspended in groundnut oil. The blood samples were collected from birds after 14 and 28 days of oral administration and analyzed for hematological and biochemical parameters. The study showed that hematological parameters [hemoglobin (Hb), packed cell volume (PCV), total erythrocyte count (TEC)] remained unaffected except total leukocyte count which was decreased at the highest dose tested only on 28^th^ day of experiment in birds of group I3. Imidacloprid produced hypoglycemia during the entire period of study, which was dose dependent. Imidacloprid treated birds showed significant increase in serum glutamate oxaloacetate transaminase (SGOT) level at 14 and 28 days of experiment, while no significant change in serum glutamate pyruvate transaminase (SGPT), serum total protein, serum total albumin, serum total globulin and serum creatinine was seen.

## INTRODUCTION

Imidacloprid, a relatively new, systemic insecticide related to nicotine, was introduced to the market in 1991 as the first chloronicotinyl insecticide, and has since become the most successful, highly effective and largest selling insecticide worldwide.[1] It has higher selectivity factors for insect versus mammals than organophosphates, methylcarbamates and organochlorines. This is attributable to both target site specificity[2] and detoxication. Due to the favorable mammalian safety characteristics, imidacloprid has been developed for veterinary use and is used as flea control agent on cats and dogs. In agriculture, it is most commonly used on rice, cereal, maize, potatoes, vegetables, sugar beets, fruits, cotton, hops and turf for control of sucking insects, coleoptera (beetles) and others.[3] Alterations in hematobiochemical parameters following intraperitoneal administration in rats[4] and oral administration in cockerels[5] have been reported. The present study was conducted to investigate the effect of repeated (28 day) oral administration of imidacloprid on hematobiochemical profiles in male White Leghorn (WLH) chicks.

## MATERIALS AND METHODS

### Experimental animals

The present study was conducted on day-old healthy male WLH chicks. The chicks were procured and housed in pens of battery brooder house at Central Poultry Research Station, Anand Agricultural University, Anand. The chicks were provided with standard feed and water *ad libitum*.

### Experimental design

Birds were acclimatized for a period of 1 week before the start of oral dosing with imidacloprid. All the birds were randomly divided into five groups (C1, C2, I1, I2 and I3), with each group containing 25 birds. Apparent LD_50_ (ALD_50_) of imidacloprid (50 mg/kg) was taken into consideration for calculation of different doses in the groups.[5] Birds were treated with imidacloprid at a dose rate of 1/20^th^ of LD_50_, 1/30^th^ of LD_50_ and 1/40^th^ of LD_50_ in three treatment groups for a period of 28 days starting from 7 days of age. Group C1 was given no treatment and served as control. The group C2 was administered groundnut oil and served as vehicle control. Group I1 was given 1/40^th^ of LD_50_ (1.25 mg/kg), group I2 was put on 1/30^th^ of LD_50_ (1.67 mg/kg) and group I3 received 1/20^th^ of LD_50_ (2.5 mg/kg) of imidacloprid suspended in groundnut oil.

The daily oral administration was continued for 28 days and the live weight was recorded at weekly intervals and birds were observed for any toxicity symptoms during the entire period of experiment.

On 7^th^ day of experiment, the birds were vaccinated with New Castle disease Lasota strain vaccine (Indovax, Gurgaon, India) through intraocular route. The birds were also vaccinated against Infectious Bursal disease (IBD) using (Georgia strain, Indovax) at day 14. Birds were vaccinated with Marek’s Disease Bivalent vaccine (Merial, Singapore) at day-old age.

After 14 and 28 days of oral administration, the birds were weighed and blood samples were collected before final culling of birds for estimating hematological parameters namely hemoglobin (Hb), packed cell volume (PCV), total erythrocyte count (TEC) and total leukocyte count (TLC), and serum biochemical parameters, namely, serum glutamate pyruvate transaminase (SGPT), serum glutamate oxaloacetate transaminase (SGOT), creatinine (CRT), total protein (TP), serum albumin, serum glucose and serum globulin using standard kits (Coral Clinical Systems, Goa, India).

## RESULTS AND DISCUSSION

The present study was conducted to see the effect of various doses of imidacloprid on the hematobiochemical profile of WLH cockerels. An approximate LD _50_ of imidacloprid, that is, 50 mg/kg b. wt. in birds, was taken into consideration for the calculation of doses of imidacloprid to be administered to cockerels.[6] The effect of administration of imidacloprid at the rate of 1/20^th^ of LD_50_ (1.25 mg/kg b. wt.), 1/30^th^ of LD_50_ (1.67 mg/kg b. wt.) and 1/40^th^ of LD_50_ (2.5 mg/kg b. wt.) once daily for 28 days on body weight, hematological parameters, carbohydrate metabolism, protein metabolism, liver and kidney functions have been investigated in the present study. Transaminases were measured to find out the effect on hepato-biliary system while CRT was measured to evaluate kidney function. TP, total albumin and total globulin were measured to see the effect of imidacloprid on protein metabolism, and serum glucose was measured for monitoring carbohydrate metabolism.

There were no apparent clinical signs of toxicity at all the dose levels tested. Besides, there was no significant effect on body weight of the imidacloprid treated birds, giving no indication of stress on the birds due to given doses of imidacloprid. The administration of imidacloprid did not create any significant change in the levels of Hb, PCV and TEC in WLH cockerels after 14 and 28 days of imidacloprid exposure; however, after 28 days of exposure, there was a significant reduction in TLC [Table 1] in the birds of group I3 (2.5 mg/kg). It has been suggested that compounds having benzene ring or other ring structure act as a hapten that combines with the protein constituent of leukocytes to form an antigen to which the animal develops antibodies that are toxic to leukocytes, causing either lysis or agglutination.[7] Imidacloprid is also a ring-structured compound and thus may have caused leukocytopenia.

**Table 1:** Effect of daily oral administration of imidacloprid on hematological parameters in White Leghorn cockerel chicks

Group	Hb (mean±SE) (g%))		PCV (mean±SE) (%)		TEC (mean±SE) (10^6^/mm^3^)		TLC (mean±SE) (/mm^3^)	
	14 days	28 days	14 days	28 days	14 days)	28 days	14 days	28 days
Group C1 (control)	9.97±62	8.47±24	29.33±1.02	30.000±73	2.57±08	2.67±08	10,167±530	10,600±520
Group C2 (vehicle control)	9.50±0.71	8.23±0.25	30.00±1.48	29.67±0.95	2.47±0.07	2.57±0.07	10,167±458	10,158±505
Group I1 (LD*50*/40)	9.27±0.46	8.23±0.25	29.33±0.33	28.50±0.62	2.52±0.11	2.67±0.11	10,083±542	10,333±487
Group I2 (LD*50*/30)	9.13±0.55	8.17±0.16	28.00±0.68	28.33±1.08	2.47±0.07	2.57±0.07	10,000±507	9883±399
Group I3 (LD*50*/20)	8.47±0.12	7.97±0.18	28.00±0.73	27.83±1.60	2.47±0.12	2.57±0.12	9967±577	8667±154*

*n* = 25;

* *P* ≤ 0.05

After 14 and 28 days, imidacloprid treated birds at different doses showed a significant dose-related increase in SGOT level as compared to controls [Table 2]. The level being highest in birds of group I3, the elevation of SGOT may be due to degenerative pathological changes in hepatocytes, which cause increase in permeability of cell membrane, resulting in release of transaminases in the blood stream.[8] Similar increase in SGOT values by imidacloprid has been reported in cockerels.[5] While imidacloprid treated birds at different doses did not show any significant change in SGPT level, SGPT activity is of little diagnostic value in diseases of birds.[9] There was no significant effect on serum TP, albumin, globulin and CRT levels, indicating no effect on protein metabolism and kidney function due to administered doses of imidacloprid. These are in agreement with the findings of Premlata *et al*.[4] in rats.

**Table 2:** Effect of daily oral administration of imidacloprid on biochemical parameters in White Leghorn cockerel chicks

Group	SGOT (U/l) (mean±SE)		SGPT (U/l) (mean±SE)		Serum total protein (g/dl) (mean±SE)		Serum albumin (g/dl) (mean±SE)		Serum globulin (g/dl) (mean±SE)		Serum glucose (mg/dl) (mean±SE)		Serum creatinine (mg/dl) (mean±SE)	
	14 days	28 days	14 days	28 days	14 days	28 days	14 days	28 days	14 days	28 days	14 days	28 days	14 days	28 days
Group C1(control)	160.77±2.57	160.82±5.52	23.26±0.67	24.94±1.54	2.62±0.20	3.51±0.34	0.97±0.07	0.91±0.15	1.65±0.25	2.60±0.22	194.97±5.60	161.20±4.36	0.290±0.007	0.415±0.003
Group C2(vehicle control)	159.88±3.6	162.40±3.96	23.71±0.84	24.05±1.81	2.73±0.23	2.84±0.34	0.87±0.09	0.76±0.11	1.83±0.17	1.91±0.29	193.21±5.17	150.48±3.70	0.300±0.008	0.409±0.005
Group I1(LD_50_/40)	183.07±2.82	202.17±8.33*	21.62±1.64	22.88±1.10	2.28±0.06	3.46±0.15	0.85±0.13	1.02±0.05	1.42±0.13	2.43±0.16	191.216±4.64	110.23±6.46**	0.298±0.008	0.409±0.005
Group I2(LD_50_/30))	193.70±5.12	227.02±8.93**	21.50±1.06	22.10±0.95	2.33±0.04	3.53±0.23	0.87±0.15	1.06±0.21	1.46±0.13	2.47±0.35	201.27±5.05	102.47±4.93*	0.307±0.003	0.401±0.008
Group I3(LD_50_/20)	214.03±8.58	237.47±9.15**	23.50±0.93	25.22±1.73	2.29±0.23	2.99±0.18	0.64±0.08	1.13±0.09	1.65±0.27	1.95±0.17	169.40±6.03**	98.34±5.53**	0.302±0.006	0.387±0.011

n = 25;

* *P* ≤ 0.05

** *P* ≤ 0.01

At 14 days, imidacloprid treated birds at highest dose (I3) showed a marked decrease in glucose levels, while there was no significant effect on birds of groups I1 and I2. However, after 28 days of exposure, there was a significant decrease in glucose level in all the insecticide treated groups. The birds in group I3 exhibited the highest decrease in glucose levels. Hypoglycemia induced by the given doses of imidacloprid may be due to the fact that thyroid is especially sensitive to imidacloprid. In 1998, an US EPA report indicated that imidacloprid can affect thyroid function in animals, which may cause decrease in blood glucose levels.

In conclusion, imidacloprid seems to be less toxic for multiple systems in WLH chicks at the doses administered. However, dose-dependent decrease in blood glucose and increased SGOT indicative of degenerative changes in liver were caused by imidacloprid. Besides, imidacloprid caused leukocytopenia at the dose of 2.5 mg/kg/day.
